# Biochemical Characterization, Action on Macrophages, and Superoxide Anion Production of Four Basic Phospholipases A_**2**_ from Panamanian *Bothrops asper* Snake Venom

**DOI:** 10.1155/2013/789689

**Published:** 2012-12-24

**Authors:** Aristides Quintero Rueda, Isela González Rodríguez, Eliane C. Arantes, Sulamita S. Setúbal, Leonardo de A. Calderon, Juliana P. Zuliani, Rodrigo G. Stábeli, Andreimar M. Soares

**Affiliations:** ^1^Departamento de Análises Clínicas, Toxicológicas e Bromatológicas, Faculdade de Ciências Farmacêuticas de Ribeirão Preto, Universidade de São Paulo, FCFRP-USP, Ribeirão Preto, SP, Brazil; ^2^Departamento de Química, Facultad de Ciencias Naturales y Exactas, Universidad Autónoma de Chiriquí, UNACHI, Panama; ^3^Departamento de Laboratorio Clínico, Complejo Hospitalario Metropolitano, Caja de Seguro Social, CHM-CSS, Panama; ^4^Departamento de Física e Química, Faculdade de Ciências Farmacêuticas de Ribeirão Preto, Universidade de São Paulo, FCFRP-USP, Ribeirão Preto, SP, Brazil; ^5^Centro de Estudos de Biomoléculas Aplicadas a Saúde-CEBio, Fundação Oswaldo Cruz, FIOCRUZ Rondônia e Núcleo de Saúde, Universidade Federal de Rondônia, UNIR, Porto Velho, RO, Brazil

## Abstract

*Bothrops asper* (Squamata: Viperidae) is the most important venomous snake in Central America, being responsible for the majority of snakebite accidents. Four basic PLA_2_s (pMTX-I to -IV) were purified from crude venom by a single-step chromatography using a CM-Sepharose ion-exchange column (1.5 × 15 cm). Analysis of the N-terminal sequence demonstrated that pMTX-I and III belong to the catalytically active Asp49 phospholipase A_2_ subclass, whereas pMTX-II and IV belong to the enzymatically inactive Lys49 PLA_2_s-like subclass. The PLA_2_s isolated from Panama *Bothrops asper* venom (pMTX-I, II, III, and IV) are able to induce myotoxic activity, inflammatory reaction mainly leukocyte migration to the muscle, and induce J774A.1 macrophages activation to start phagocytic activity and superoxide production.

## 1. Introduction

Envenomation resulted from snakebites is the cause of considerable morbidity and mortality in many tropical and subtropical countries, being an important public health problem [1, 2]. According to Kasturiratne et al. [2], the estimative number of deaths per year due to snakebites in 2007 for Central America ranged from 193 to 1,461. Since snakebites affect, in most cases, poor people living in rural parts of tropical countries [3], the World Health Organization (WHO), incorporated snakebite envenoming in its list of neglected diseases (http://www.who.int/neglected_diseases/diseases/en/).

**Figure 1:**
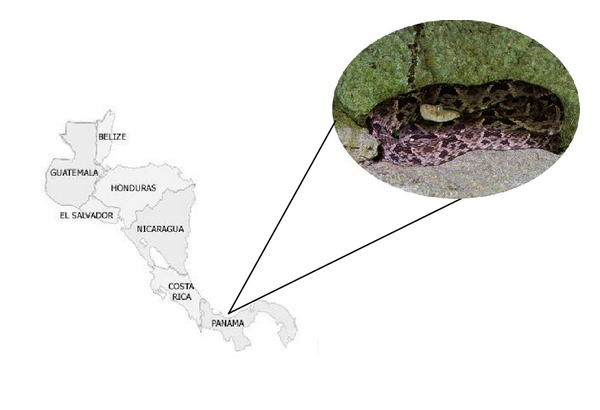
*Bothrops asper *resting in limestone cave, Central Panama.

Lance-headed pit vipers belonging the Viperidae family, especially the *Bothrops asper *snake (Figure 1) are responsible for the most severe cases of snakebite envenoming, being the main cause of the largest numbers of bites and fatalities in Central America [1]. Among the American countries, Panama has thehighest incidence of snakebite cases, showing an average number of 40 to 65 cases per 100,000 population per year and a estimated total number of 1,300 to 1,800 cases per year [4, 5], which *B. asper* is responsible for 90% of all snakebites cases of major clinical importance [4, 6]. *B. asper* is able to inoculate a relative large amount of venom and is considered extreme aggressive, being able to cause severe accidents [7].

According to Gutiérrez et al. [5], the envenomation produced by *B. asper* induces prominent local tissue damage, characterized by swelling, blistering, prominent oedema, haemorrhage, dermonecrosis, and myonecrosis with clinical manifestations that include bleeding, effects on platelet aggregation, coagulopathy, hypotension, hemodynamic alterations, pulmonary oedema, and acute renal failure. Other less common effects include intravascular haemolysis, acute myocardial damage multiple organ failure, and death. The clinical features of the envenomation are affected by the venom components, which vary according to snake species, geographic region, age, sex, and environment [5, 8, 9]. 

Snake venoms are characterized as a complex mixture of bioactive molecules, which proteins compose more than 90% of the venom dry weight [10–12]. Many of these proteins are enzymes, in which the most abundant are phospholipases A_2_ (PLA_2_s; E.C.3.1.1.4) [10]. PLA_2_s are members of a protein superfamily that comprise several groups of enzymes with different catalytic mechanisms, as well as different functional and structural features, that cleavage the sn-2 acyl ester bond of glycerolphospholipids producing free fatty acids and lysophospholipids [13, 14]. Snake venom PLA_2_s (svPLA_2_s) have been grouped into four classifications according to minor structural differences as group I and II, both sub-classified as type A or B. The group II is found in venoms from Viperidae family, while the group I is found in Elapidae and Hydrophiidae venoms [14]. svPLA_2_s from Viperidae family are placed into group IIB and are mainly subdivided in two types: Asp49 PLA_2_s, which have an Asp residue at position 49, and Lys49 PLA_2_s, showing a Lys residue at position 49. Different from Asp49 PLA_2_s, Lys49 PLA_2_s have low or any catalytic activity upon artificial substrates [13, 15, 16].

This present paper describes the biochemical and toxicological characterization of crude *B. asper *venom from Panama, and the isolation, purification, and biochemical characterization of four basic cytotoxic PLA_2_ from this venom and its effects on gastrocnemius muscle and inflammation.

## 2. Materials and Methods

### 2.1. Materials

*Bothrops asper* snake venom was collected from adult specimens, captured in Caldera and Gomez (Provence of Chiriquí, Panama) and in Arraiján (Provence of Panama, Panama). The snakes were maintained in a serpentarium at the Gamboa Rainforest Resort, Panamá, where the crude venom was obtained by inducing the snake to bite a parafilm-wrapped jar. Venoms were centrifuged at 1,000 xg for 15 min, and supernatants were lyophilized and stored at −20°C in Microbiology Department at the Medicine Faculty of Panama University until used. Male albino Swiss mice, weighing 18–22 g, were used for the assays.

The murine macrophage cell lines (J774A.1) were obtained from Rio de Janeiro Cell Bank Collection (Brazil). RPMI-1640, penicillin, streptomycin, and L-glutamine were purchased from Sigma-Aldrich (MO, USA); fetal bovine serum (FBS) was from Cultilab (Brazil). All reagents were low endotoxin or endotoxin-free grades. Animal care was in accordance with the guidelines of the Brazilian College for Animal Experimentation (COBEA) and was approved by the Committee for Ethics in Animals Utilization of Universidade de São Paulo (CEUA no. 06.1.291.53.3). CM-Sepharose and Phenyl-Sepharose resins were purchased from Amersham Biosciences, Uppsala, Sweden. The Kit CK-UV- Kinetic was purchased from Bioclin, Brazil. The following reagents: ethylenediaminetetraacetic acid (EDTA), molecular weight protein standards, and acrylamide were obtained from Sigma Chemical Co. All other chemicals reagents were of analytical grade from Merck, Aldrich or Pharmacia Biotech.

### 2.2. Purification and Biochemical Characterization of PLA_2_s

*B. asper *crude venom (300 mg) was dissolved in 1.5 mL of 0.05 M ammonium bicarbonate buffer, pH 8.1 and applied on a CM-Sepharose column (1.5 × 15 cm) according to Soares et al. [17]. All fractions were analyzed by SDS-PAGE, and PLA_2_ activity was evaluated *in vitro* by indirect erythrocyte lysis in agar containing human erythrocytes and egg yolk, as previously described [18], being the fraction with PLA_2_ activity selected. Polyacrylamide gel electrophoresis was performed in the presence of sodium dodecyl sulfate (SDS-PAGE) [19]. Isoelectric focusing was performed according to previously described. Buffalyte, pH range 3.0–9.0 (Pierce, IL), was used to generate the pH gradient. To determinate the protein concentration, the microbiuret method was used. The mol. wt of PLA_2_s was estimated by mass spectrometry (Quattro II, Micromass). A Procise-491 (Applied Biosystems) automatic sequencer was used for the N-terminal sequencing [17]. The phenylthiohydantoin (PTH) amino acids were identified by comparing their retention times with the 20 PTH-amino acid standard mixture. The peptides obtained were compared with the sequences of other related proteins in the SWISS-PROT/TrEMBL databases using the FASTA and BLAST tools.

### 2.3. Biological and Pharmacological Characterization

#### 2.3.1. Lethality

Groups of four mice (18–22 g) were injected by IP route with various amounts of crude venom (in a volume of 0.1 mL) eluted with PBS, (phosphate buffered saline, 0.12 M NaCl, 0.04 M Na_2_HPO_4_, and pH 7.2). Deaths were recorded at 1, 3, 6, 12, 24, and 48 hours. LD_50_ was calculated using the Spearman-Karber method [20].

#### 2.3.2. Edema-Inducing Activity

 Groups of four male Swiss mice (18–22 g) were injected in the subplantar region with various amounts of crude venom (in a volume of 50 *μ*L) prepared with PBS, pH 7.2. Then, the paw increase was measured at different time intervals (30, 60, 120, and 180 min), subtracting the initial paw measure (time 0 h). The paw edema was measured with the aid of a low-pressure pachymeter (Mitutoyo, Japan).

#### 2.3.3. Hemorrhage

Groups of four male Swiss mice (18–22 g) were injected by ID route in the dorsal region with various amounts of crude venom (in a volume of 50 *μ*L) prepared in PBS, pH 7.2. The hemorrhagic activity of the venom was determined as follows. Different doses of venom were injected intradermally, in a volume of 0.1 mL, into groups of four mice (18–22 g); 2 h later, they were sacrificed with CO_2_, their skin removed, and the area of the hemorrhagic spot was measured. Diameters were calculated, and the minimum hemorrhagic dose was defined as the dose of venom, which induced a lesion of 10 mm diameter [21].

#### 2.3.4. Coagulant Effect

 Platelet-poor plasma was obtained from rabbit citrated blood by centrifuging the plasma twice at 2,500 xg for 15 min at 4°C. Aliquots of 0.5 mL of platelet-poor plasma were incubated with various amounts of crude venom (dissolved in 100 *μ*L of PBS, pH 7.2). Incubation was carried out for 5 min at 37°C. Then, 0.1 mL of 0.25 M CaCL_2_ was added to each tube, and they were checked for the formation of a clot every 30 seconds for a total period of 2 h. All experiments were carried out in triplicate.

#### 2.3.5. Fibrinolytic Activity

The fibrinolytic activity was measured using 0.6% bovine plasminogen-free fibrin plates [22]. For this purpose, 30 *μ*L of sample was placed on a fibrin plate, and the lysis area was measured after incubation at 37°C for 18 h. PBS was used as negative control. The specific activity was calculated from a standard curve for the lysis area obtained with plasmin on the plasminogen-free fibrin plates. All experiments were carried out in triplicate.

#### 2.3.6. PLA_2_ Activity

It was determined by incubating 0.5 mL of crude venom solution (at various amounts) with 50 *μ*L of egg yolk diluted 1 : 5 with 0.1 M Tris, 10 mM CaCl_2_, and pH 8.5 buffer containing 1% Triton X-100. Incubations were carried out for 10 min at 37°C. The liberated free fatty acids were extracted and titrated according to the method of Dole [23]. Crude venom from *Bothrops asper* (10 *μ*g) and PBS were used as positive and negative controls, respectively. All experiments were carried out in triplicate.

#### 2.3.7. Myotoxic Activity

Groups of four male Swiss mice (18–22 g) were injected in the right gastrocnemius muscle with crude venom (50 *μ*g/50 *μ*L of PBS), MTXs (50 *μ*g/50 *μ*L of PBS), or PBS alone (50 *μ*L). After 3 h, blood was collected from the tail in heparinized capillary tubes and centrifuged for plasma separation. Activity of creatine kinase (CK) was then determined using 4 *μ*L of plasma, which was incubated for 3 min at 37°C with 1.0 mL of the reagent according to the kinetic CK-UV protocol from Bioclin, Brazil. The activity was expressed in U/L, where one unit corresponds to the production of 1 mmol of NADH per minute.

#### 2.3.8. Histological Examination of Myonecrosis

Myotoxic activity was assayed on the basis of the morphologic alterations induced by IM injections of crude venom or MTXs (50 *μ*g) and negative control PBS (50 *μ*L) in the right gastrocnemius muscle of Swiss mice (18–22 g, *n* = 4). After 24 h, the animals were euthanized with CO_2_, and a small section of the central region of the muscle was excised and soaked in fixing solution (10% formaldehyde in PBS, v/v). The material was then dehydrated by increasing concentrations of ethanol and processed for inclusion in paraffin. The resulting blocks were sliced in 2.5 *μ*m thick sections, stained with 0.25% (w/v) hematoxylin-eosin and examined under a light microscope [17].

#### 2.3.9. Cytotoxic Assay

Cell viability was measured by Trypan blue exclusion. In brief, monolayers of J774A.1 cells grown in RPMI-1640 medium with 100 *μ*g/mL penicillin, 100 *μ*g/mL streptomycin, and 2 mM L-glutamine were withdrawn and after counting 2 × 10^5^ cells/80 *μ*L were added to plastic vials and incubated with 20 *μ*L of different concentrations of pMTX-I, II, and III (1.5, 3 and 6 *μ*g/mL) diluted in RPMI (control), for 1 h at 37°C in a humidified atmosphere (5% CO_2_). Then, 20 *μ*L 0.1% Trypan blue was added to 100 *μ*L of J774A.1 macrophage suspension. Viable cell index was determined in a Neubauer's chamber by counting a total number of 100 cells. Results were expressed as percentage of viable cells. 

#### 2.3.10. Colorimetric NBT Assay

The colorimetric NBT assay was conducted in J744A.1 cells. In this assay, the generation of superoxide was estimated by reducing nitroblue tetrazolium (NBT), a yellow liposoluble compound that becomes insoluble and blue in its reduced form [24]. For this test, the cells J774A.1 had their concentration adjusted to 2 × 10^5^/100 *μ*L and were incubated with 100 *μ*L of RPMI containing NBT 0.1% (control) or 100 *μ*L of 2 × 10^6^ zymosan particles suspension diluted in RPMI containing NBT 0.1% (positive control) or 100 *μ*L of different concentrations of pMTX-I, II, and III (1.5, 3 and 6 *μ*g/mL) diluted in RPMI containing NBT 0.1%, and incubated for 1 h at 37°C in humidified atmosphere (5% CO_2_). At the end of the incubation period, the vials were centrifugated for 30 seconds at 4,500 xg, and the cells were washed twice with warm PBS. The NBT reduced deposited inside the cells were then dissolved, first by adding 120 *μ*L of 2 M KOH to solubilize cell membranes and then by adding 140 *μ*L of DMSO to dissolve blue formazan with gentle shaking for 10 min at room temperature. The dissolved NBT solution was then transferred to a 96-well plate and absorbance was read on a microplate reader at 620 nm. Data were expressed as absorbance.

#### 2.3.11. Phagocytic Activity of J774A.1 Cells of Nonopsonized Zymosan

J774A.1 cells were plated on 13 mm diameter glass coverslips (Glass Tecnica, Brazil) in 24-well plates at a density of 2 × 10^5^ cells per coverslip and allowed to attach for 2 h at 37°C under a 5% CO_2_ atmosphere. Nonadherent cells were removed by washing with PBS. Cell monolayers were cultured for 1 h with RPMI supplemented with 100 *μ*g/mL penicillin, 100 *μ*g/mL streptomycin, and 2 mM L-glutamine at 37°C and 5% CO_2_, and then challenged with RPMI (control) or 6 *μ*g/mL of pMTX-I, II, and III diluted in RPMI. After washing in cold PBS, the monolayers were incubated for 1 h at 37°C and 5% CO_2_ with nonopsonized zymosan, prepared as described below, and unbound particles were removed by washing with PBS. Cells were fixed with 2.5% glutaraldehyde for 15 min at room temperature, and the coverslips were mounted in microscope slides. The extent of phagocytosis was quantified by contrast phase microscopic observation. At least 200 macrophages were counted in each determination, and those containing three or more internalized particles were considered positive for phagocytosis [25, 26]. Results were presented as the percentage of cells positive for phagocytosis.

The zymosan particles, obtained from yeast cell walls, were suspended in PBS providing a concentration of 3 mg/mL. After that, the zymosan suspension was sonicated for 15 min, and total zymosan particles were determined in a Neubauer's chamber. The ratio of zymosan per macrophage was 1 : 10.

### 2.4. Statistical Analysis

Results are presented as mean ± S.D. obtained with the indicated number of tests. The statistical significance of differences between groups was evaluated using *t* student test. A 0.1 < *P* < 0.05 value was considered to indicate significance.

## 3. Results and Discussion

Panamanian *B. asper* snake venom induced hemorrhage, edema, myonecrosis, coagulation, and fibrinolytic activities *in vitro*, and lethality, also presenting PLA_2_ activity evidenced by titrimetric and indirect hemolytic assays (Table 1). The toxicological profile was qualitatively similar to that previously described for *B. asper* from Costa Rica [12] and Guatemala [27]. The nature and biological properties of snake venom components are peculiar to each species [8], whereas the presence and concentration of several venom components could vary intraspecifically as a function of geographic distribution, age, sex, feeding, size, season, and the time elapsed between venom extraction [9, 11, 28]. These intraspecific variations have evident clinical and therapeutic implications and can affect the capacity of antivenoms to neutralize venoms from snakes of geographically separated populations [5, 11, 13, 29, 30].

**Figure 2:**
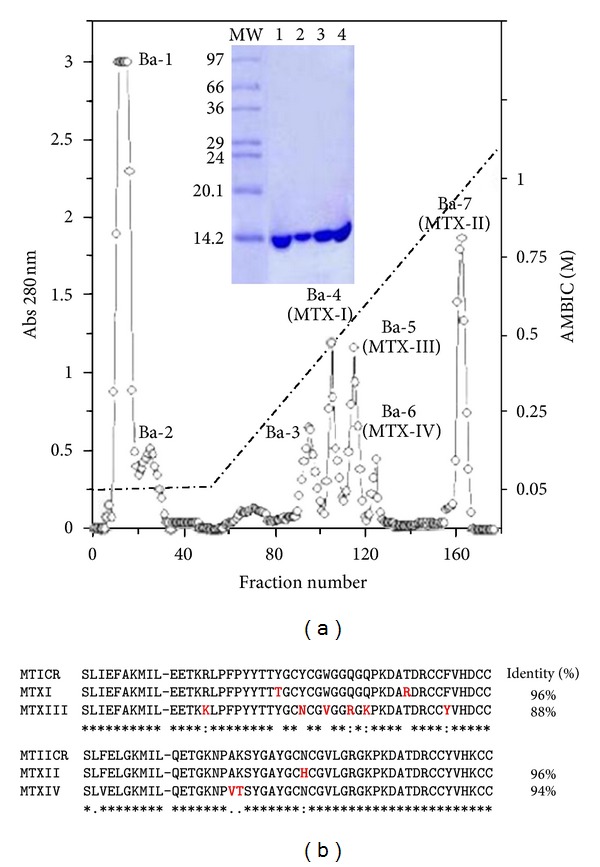
(a) Purification of myotoxins: ion exchange column of Panama *B. asper *venom (300 mg) on a CM-Sepharose column equilibrated with AMBIC 0.05 M pH 8.0 and eluted with a concentration gradient of AMBIC up to 1 M at a flow rate of 1.5 mL/minute. Inserted: SDS-PAGE 12%. Samples: MW (molecular weight markers); (1) pMTX-I (20 *μ*g); (2) pMTX-II (20 *μ*g); (3) pMTX-III (20 *μ*g); (4) pMTX-IV (20 *μ*g). (b) Comparison of the N-terminal amino acid sequence of phospholipases A_2_ isolated from Panama *B. asper *venom: pMTX-I and III belong to the subgroup Asp49, whereas pMTX-II and IV belong to the subgroup Lys49 when compared with myotoxin III (P20474-PA21 BOTAS) Asp49 and myotoxin II (P24605-PA2H2 BOTAS) Lys49 from Costa Rica *B. asper *venom.

**Figure 3:**
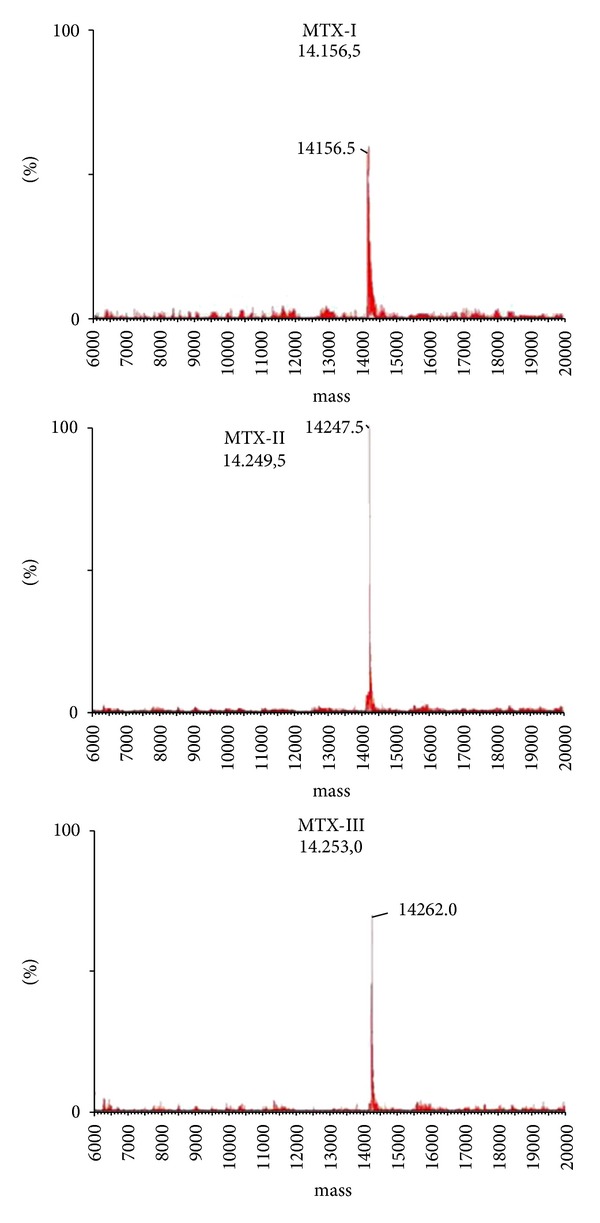
Analysis of mass spectra of PLA_2_s isolated: pMTX-I, Mr = 14,156; pMTX-II, Mr = 14,249; pMTX-III, Mr = 14,253.

Four basic svPLA_2_s were highly purified in a single step purification using an ion-exchange chromatography performed on a CM-Sepharose column. The elution of absorbed proteins with a linear gradient of concentrated buffer resulted in seven fractions (Figure 2(a)), which fractions Ba-4 to Ba-7 were related to PLA_2_s. Ba-4 and Ba-5 fraction were related to PLA_2_ enzymatically active, whereas Ba-6 and Ba-7 were related to enzymatically inactive PLA_2_. The purity degree of the isolated proteins was further demonstrated by SDS-PAGE, mass spectrometry, and N-terminal sequence (Figures 2 and 3) and named as pMTX-I, pMTX-III, pMTX-IV, and pMTX-II, respectively. Different from our study, others research groups have exhaustively purified PLA_2_s from *Bothrops* venoms using different combinations of chromatographic methods: gel filtration, ion exchange, RP-HPLC, and affinity with antibodies and heparin [13, 31]. 

**Figure 4:**
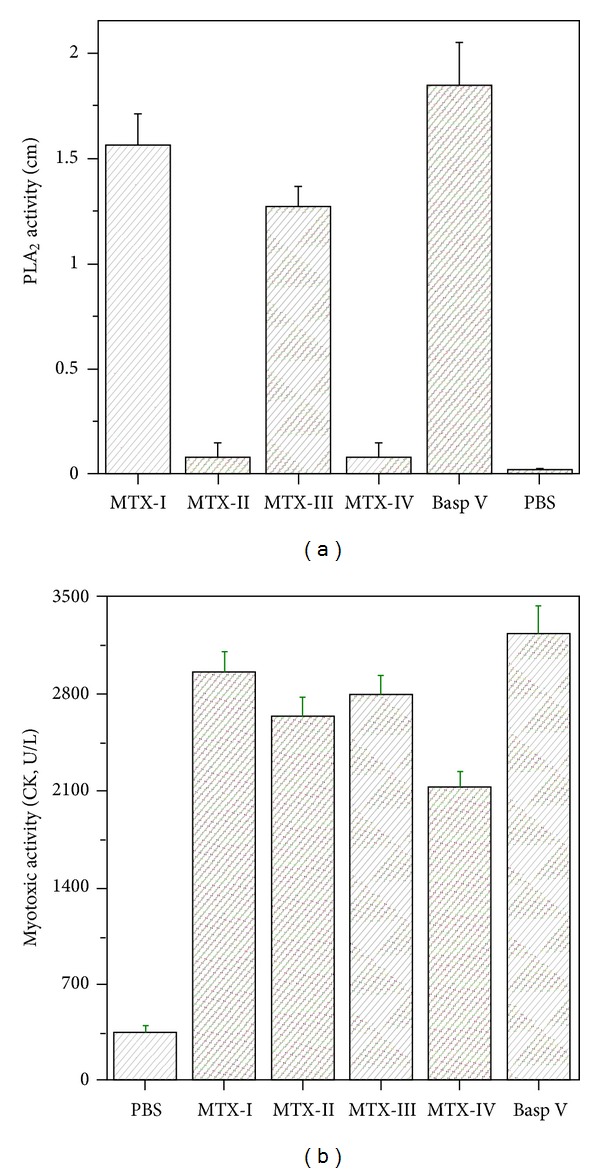
(a) Phospholipase activity (indirect hemolysis) of the isolated enzymes from Panama *B. asper *venom. Samples: pMTX-I (5 *μ*g); pMTX-II (10 *μ*g); pMTX-III (5 *μ*g); pMTX-IV (10 *μ*g). Negative control (PBS) and positive control (VBasp, *B. asper *venom, 10 *μ*g); (b) myotoxic activity of the PLA_2_s isolated from Panama *B. asper *venom. *Swiss *mice were injected with 50 *μ*L of the different samples in the right gastrocnemius muscle and, after 3 h, the blood from the tail was collected in capillaries with heparine. Negative (PBS), positive control (BaspV, *B. asper *venom, 25 *μ*g), and isolated myotoxins (50 *μ*g) were used. Values represent the mean ± S.D. from 3 independent experiments.

The purified proteins were characterized as single polypeptide chains, with an isoelectric point ranging from 8.1 to 8.3. The average molecular mass estimated by mass spectrometry was 14,156.5 for pMTX-I, 14,249.5 for pMTX-II, and 14,253.0 for pMTX-III (Figure 3). The N-terminal sequence alignment of pMTX-I and pMTX-III with MTICR myotoxic III PLA_2_ (Uniprot accession no.: P20474) from Costa Rican *B. asper *showed, respectively, 96% and 88% of identity, and the sequence alignment of pMTX-II and pMTX-IV showed, respectively, 96% and 94% of identity with *B. asper* MTIICR myotoxic IV PLA_2_ (Uniprot accession no.: P24605). Additionally, multiple sequence alignment of pMTX-I, II, III, and IV with Costa Rica *Bothrops* PLA_2_s showed highly conserved amino acids, such as cysteine residues involved in disulfide bond formation. Several other conserved residues important to PLA_2_ catalytic activity, such as the catalytic site (D_42_XCCXXHD_49_) and the calcium-binding site (X_27_CGXGG_32_) [13, 31] were shown. The N-terminal sequences (Figure 2(b)) of the isolated MTXs demonstrated that pMTX-I and III are basic PLA_2_s with an aspartate residue at position 49 (Asp49), therefore catalytically active (Figure 4(a)), whereas MTX-II and IV are basic PLA_2_s displaying a lysine residue at the same position (Lys49) (Figure 4(a)), therefore, catalytically inactive. 

Panamanian *B. asper* crude venom, pMTX-I, II, III, and IV showed a high myotoxic activity (Figure 4(b)). Histopathological analysis revealed a drastic myonecrosis, displaying contracted and clumped fibers in different stages of degeneration and leukocyte infiltrate induced by myotoxins. Our results agree with Gutiérrez and Lomonte [32] and suggest that Lys49 myotoxins pMTX-II and pMTX-IV can affect the cell membrane of skeletal muscle fibers by a phospholipid hydrolysis independent mechanism. These results suggest that, moreover the catalytic site, this toxin may possess another molecular region that can bind and disorganize skeletal muscle plasma membrane [31, 32]. Some studies have suggested that myotoxic PLA_2_s may induce muscle cell damage by affecting the integrity of plasmatic membranes, thereby leading to hyper contraction and other intracellular effects [13, 31, 32].

**Figure 5:**
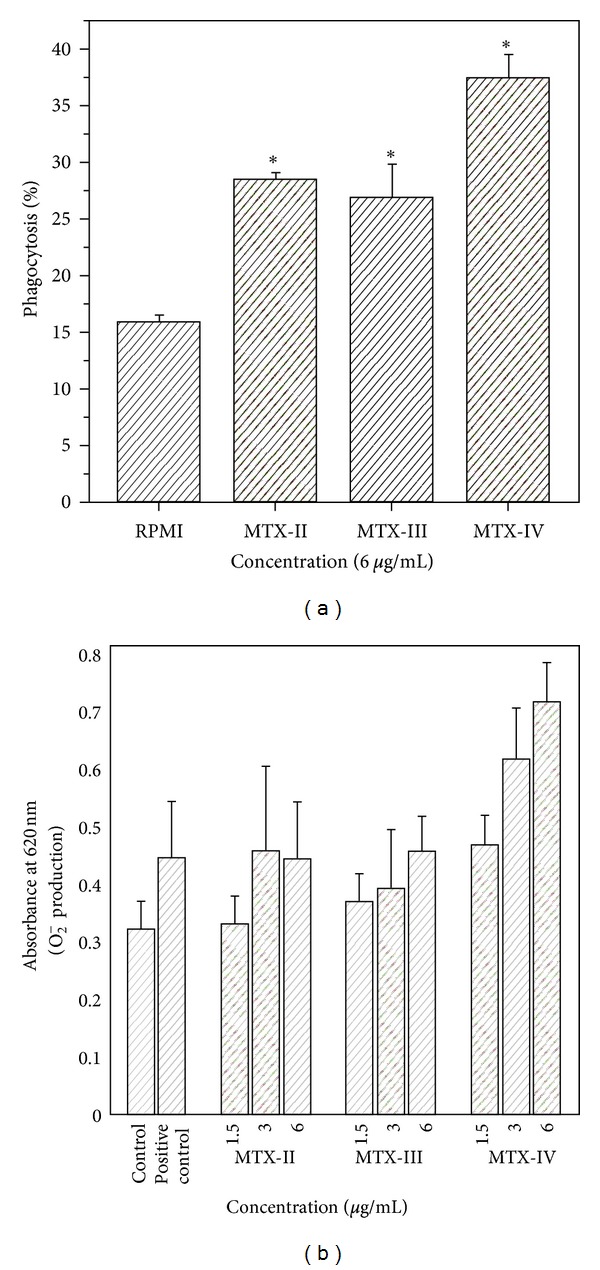
Effect of pMTX-II, pMTX-III, and pMTX-IV on phagocytosis (a) and O_2_^−^ production (b) by J774A.1 macrophages. The phagocytosis of nonopsonized zymosan particles was determined by phase-contrast microscopy. J774A.1 macrophages were incubated with 6 *μ*g/mL of pMTX-II, pMTX-III,pMTX-IV, and RPMI (control) during 60 minutes before addition of non-opsonized zymosan particles. Values represent the mean ± S.D.M. from 3–5 independent experiments. *P < 0.05 compared with control ^#^ *P* < 0.05 compared with positive control (ANOVA).

 In order to evaluate the activation of leukocytes, the toxicity of *B. asper* myotoxins on macrophage J774A.1 cell line were studied. The cells were incubated with different concentrations of pMTX-II, III, and IV during 1 hour. These myotoxins did not affect the macrophage viability, which are in agreement with Zuliani et al. [25], showing their low toxicity on this cell type. Additionally, the effect of the same myotoxins on J774A.1 phagocytosis ability was evaluated via *β*-glucan receptor, by the uptake of nonopsonized zymosan particles incubated with noncytotoxic concentrations of pMTX-II, III, and IV was investigated. Our data showed that J774A.1 macrophages incubated with RPMI showed an average of phagocytosis of 16.5 ± 0.5%. Incubation of macrophages with pMTX-II, III, and IV, at 6 *μ*g/mL, resulted in phagocytic indexes of 30.6 ± 0.6%, 29.3 ± 5.5%, and 36.5 ± 2.5%, respectively. These results showed that the myotoxins studied were able to stimulate phagocytosis of non-opsonized zymosan particles by J774A.1 macrophages (Figure 5(a)), which are in agreement with Zuliani et al. [25]. Moreover, these results suggest that phospholipid hydrolysis catalytic activity is not essential for the activity observed and argue with the hypothesis that other molecular regions distinct from the active site may be involved in this effect.

 One of the most immediate responses of macrophages during phagocytosis is the production of the potent oxygen free radical, superoxide anion. The enzyme complex primarily responsible for the production of this highly reactive oxygen species is the NADPH oxidase complex [33]. This reaction parallels the release of a variety of inflammatory mediators that play crucial roles in the host defense by microbial killing, but may also cause injury to surrounding tissues [33–35]. In order to investigate the ability of pMTX-II, III, and IV to induce the production of superoxide by a macrophage cell line J774A.1, the cells were incubated with non-cytotoxic concentrations of myotoxins. As shown in Figure 5(b), J774A.1 macrophages incubated with RPMI (negative control) showed a superoxide production average of 0.316 ± 0.05 D.O., and J774A.1 incubated with RPMI plus non-opsonized zymosan (positive control) showed a superoxide production average of 0.455 ± 0.1 D.O. Incubation of macrophages with pMTX-II, III, and IV, at 3 and 6 *μ*g/mL, respectively, induced a significant production of O_2_^−^ in J774A.1 macrophages, showing that myotoxins are able to induce superoxide production by J774A.1 macrophages, indicating the ability of these toxins to activate these cells. Again, these results suggest that the PLA_2_ catalytic activity is not important in macrophage activation. Thus, in accordance with our results, increments in hydrogen peroxide (H_2_O_2_), another reactive oxygen specie generated by a multicomponent enzyme system, NADPH-oxidase, have been described in thioglycollate-elicited macrophages incubated with MTX-II and III from Costa Rica *B. asper* venom [25]. 

It is important to note that phagocytosis mediated by *β*-glucan receptors and also by mannose and Fc*γ* receptors are coupled to the production of both proinflammatory and microbicidal molecules, such ROS [36, 37]. Release of ROS by phagocytic cells has been implicated in microbial killing [38] as well as in the damage to host surrounding tissue [39]. Considering that MT-II and MT-III isolated from *B. asper* venom from Costa Rica display a broad cytolytic activity and affect a variety of cell types in culture [40, 41], our findings suggest the role of O_2_^−^ in the cytotoxicity induced by these myotoxins, a hypothesis that can be addressed with the use of antioxidant agents.

In conclusion, the Panamanian *B. asper* venom has qualitatively a similar toxicological profile to those previously described for *B. asper* from Costa Rica and Guatemala despite the observation of quantitative variations in these activities. The PLA_2_s isolated from Panama *Bothrops asper* venom (pMTX-I, pMTX-II, pMTX-III, and pMTX-IV) induced myotoxic activity, inflammatory reaction mainly leukocyte migration to the muscle, activation of macrophages to exert phagocytic activity, and production of superoxide.

## Figures and Tables

**Table 1:** Toxic activities induced by *B. asper* snake venom from Panama.

Effect^a^	Activity
Lethal (LD_50_; *μ*g/mouse)	55
Edema-inducing (DEM; *μ*g)^ab^	1 ± 0.1
Hemorrhagic (MHD, *μ*g)^ab^	1.2 ± 0.2
Coagulant (MCD; *μ*g)^ab^	3.5 ± 0.05
Fibrinolytic (MFD; *μ*g)	0.5
Indirect hemolitic (MHeD; *μ*g)^ab ^	7.2 ± 0.01
Phospholipase A_2_ (mEq/mg·min)^ab ^	27.2 ± 0.45
Myotoxic (MMD, *μ*g)^ab^	10 ± 1

^a^Results are presented as mean ± SD. Except for lethality and fibrinolytic activity.

^b^All experiments were carried out in triplicate.
